# Microfabricating Mirror-like Surface Precision Micro-Sized Amorphous Alloy Structures Using Jet-ECM Process

**DOI:** 10.3390/mi15030375

**Published:** 2024-03-11

**Authors:** Lei Han, Pingmei Ming, Shen Niu, Guangbin Yang, Dongdong Li, Kuaile Cheng

**Affiliations:** School of Mechanical and Power Engineering, Henan Polytechnic University, Jiaozuo 454003, China; hl15639739798@163.com (L.H.); ns2019@hpu.edu.cn (S.N.);

**Keywords:** electrochemical microfabrication, jet-electrolyte electrochemical machining, amorphous alloys, mirror-like surface

## Abstract

Amorphous alloy (AA) is a high-performance metal material generally with significantly excellent mechanical and corrosion resistance properties and thus is considered as a desirable material selection for micro-scale articles. However, the microfabrication of AA still faces a variety of technical challenges mainly because the materials are too hard to process and easily lose their original properties, although at moderately high temperatures. In this study, jet-electrolyte electrochemical machining (Jet-ECM) was proposed to microfabricate the Zr-based AA because it is a low-temperature material-removal process based on the anode dissolution mechanism. The electrochemical dissolution characteristics and material removal mechanism of AA were investigated, and then the optimal process parameters were achieved based on the evaluation of the surface morphologies, surface roughness, geometrical profile, and machining accuracy of the machined micro-dimples. Finally, the feasibility was further studied by using Jet-ECM to fabricate arrayed micro-dimples using the optimized parameters. It was found that Jet-ECM can successfully microfabricate mirror-like surface AA arrayed precision micro-dimples with significantly high dimensional accuracy and geometrical consistency. Jet-ECM is a promisingly advantageous microfabrication process for the hard-to-machine AA.

## 1. Introduction

Amorphous alloys, also known as metallic glasses, are prepared by multiple-component liquid melts under the conditions of supercooled solidification. Due to the special formation conditions, amorphous alloys are metastable materials that lack the long range order of conventional crystalline metals [1]. Therefore, compared with conventional metallic materials, amorphous alloys have outstanding mechanical, physical, and chemical properties, such as high strength, high hardness, excellent wear resistance, superior corrosion resistance, lower Young’s modulus, etc. [2]. This makes them have enormous potential applications in fields including aerospace, electronics, and biomedicine [3,4,5,6]. As a result, researchers have explored a variety of processing methods to fabricate amorphous alloys. Quite a few investigators [7,8,9,10,11] demonstrated that a majority of metal features and components can be formed by using plastic forming methods because amorphous alloys are superplastic at mid-temperatures. However, due to the inherent limitations of the forming principles and working modes, it is very hard to fabricate high aspect ratio blind and closed structures with the plastic forming method. Therefore, some other fabrication methods have been developed to meet more manufacturing requirements.

These developed fabrication methods include mechanical machining (drilling, milling, etc.), laser-beam machining, electrodischarge machining, and electrochemical machining. Bakkal et al. [12,13,14], Yin et al. [15], and Liu et al. [16] have found that non-crystallization surfaces could be obtained only when high thermal conductivity cutting tools are used, but the tools are severely worn during mechanical cutting of amorphous alloys [17]. Based on this, some researchers [18,19] attempted to use the abrasive assisting water jet machining method to fabricate amorphous alloys and found that this “cold” mechanical machining method does not lead to crystallized surfaces, but it has lower process controllability. In addition, Lu et al. [20], Lin et al. [21], Yang et al. [22], and Zhu et al. [23] employed the widely used heat-based laser beam energy to process amorphous alloys and confirmed that non-crystallization surfaces could be achieved only when unconventional process parameters and conditions were used, such as high scanning speed (the selected fluences changing from 5 to 25 J/cm^2^) and low power (with a fluence of 12 J/cm^2^ and a scanning speed of 300 mm/s). Hsieh [24] and Huang [25] et al. focused on the feasibility studies of electrodischarge machining amorphous alloys, and they found that crystallization and recast layers were easily formed on the machined surface. Unlike the above-mentioned material-removal methods based on the mechanical force separation and heat erosion principle, the electrochemical machining (ECM) method removes material by the anodic dissolution mechanism occurring at room temperature. Its machinability is less affected by the mechanical properties of materials such as hardness and brittleness. The machining process does not require external mechanical force or heat, and it also generates less heat and no mechanical force. This enables ECM to have significant principle and potential process advantages in manufacturing amorphous alloy structures and parts, which has attracted intensive attention. Koza et al. [26] demonstrated the feasibility of micro-electrochemical machining Zr-based amorphous alloys (Zr_59_Ti_3_Cu_20_Al_10_Ni_8_), but a special methanolic HClO_4_ solution is needed to break down the dense passivation film of the amorphous alloy. Cole et al. [27] studied the electrochemical characteristics of Zr-based amorphous alloys (Zr_57_Ni_20_Al_15_Cu_5.5_Nb_2.5_) in a 2.98 M sodium nitrate aqueous solution, and their findings showed that the main corrosion mechanism of Zr-based amorphous alloys was pitting. They further designed a special pulse current waveform containing pitting potential and over-passivation potential of amorphous alloys to obtain better-shaped micro-pit structures. In recent years, Wu et al. [28] used the optimized electrolyte NaNO_3_-ethylene glycol to study the electrochemical properties and evaluated the effects of electrochemically micro-machining Zr-based amorphous alloys (Zr_41.2_Ti_13.8_Cu_12.5_Ni_10.0_Be_22.5_), and they found that smoother machined surfaces can be achieved by using this hybrid solution under the electrolyte-jet condition. Afterwards, they further carried out the experimentation studies on micromachining amorphous alloys using the micro-rod/wire-shaped tool electrodes. In the past decade, Zeng and Meng et al. [29,30,31,32,33,34,35,36,37,38] have carried out a series of studies on fabricating amorphous alloy microstructures by using electrochemical wire cutting and successfully achieved several complex precision microstructures such as microgear and micro-cantilever arrays [32,39]. However, electrochemical wire cutting can only be used to fabricate perforated microstructures such as slits and holes and externally complex-shaped structures by following a designed trajectory, and it is difficult to machine blind holes, grooves and cavities, and three-dimensional complex structures and parts. Furthermore, its machining efficiency is very small, and the cutting surface is subject to thermal stress.

Different from the above-mentioned electrochemical machining processes, electrolytic jet machining (Jet-ECM) is a nontraditional ECM process that uses a “cathodic” electrolytic high-speed jet to remove material and can conveniently manufacture 3D geometric structures. Compared with other types of electrochemical machining processes, Jet-ECM has the advantages of faster material-removal speed, more flexible manipulation, better spatial accessibility, and ease of automation control. Therefore, Jet-ECM has been highly used by researchers and engineers. Its fundamental theories, key technologies, and potential application development have been well studied [40,41,42]. Kawanaka et al. [39] achieved a mirror-like micro-pit structure on a stainless steel workpiece using Jet-ECM with bipolar pulse current. Bisterova et al. [41] fabricated complex micro-patterns on the workpiece surface via the numerical programming Jet-ECM. The surface roughness of the microstructures they [41] processed can reach 0.04 μm, while the surface roughness of the micro-pits obtained by conventional electrochemical processing is only 58.82 μm at the minimum [27]. Mitchell et al. [43] obtained high-precision, high-aspect-ratio microstructures with a side wall taper of 172 μm/mm by using the modified nozzle of Jet-ECM, which optimizes the current density distribution. Zhang [44] et al. studied the influence of the inclination angle of the jet nozzle on the processing quality. They concluded that when the inclination angle between the nozzle and the workpiece is 30°, the mirror structure can be generated. Nevertheless, Jet-ECM has some limitations, such as poor machining results in machining structures such as deep blind holes/slots.

Although Jet-ECM has been frequently used to fabricate stainless steel [40,41] and other metal materials [45], it has rarely been employed to process amorphous alloy materials. The high-speed electrolyte jet of Jet-ECM can facilitate the effective and efficient removal of the machined products and also can enable the process to be exerted on much higher current densities. These two factors will be remarkedly beneficial to remove the process obstacles, which unqualify the electrochemical microfabrication of amorphous alloys. Therefore, this paper aims to evaluate the feasibility of electrochemically microfabricating amorphous alloys with Jet-ECM and further discusses the electrochemical dissolution characteristics of Zr-based amorphous alloy and the machining effects of generating microstructures.

## 2. Materials and Methods

**Figure 1:**
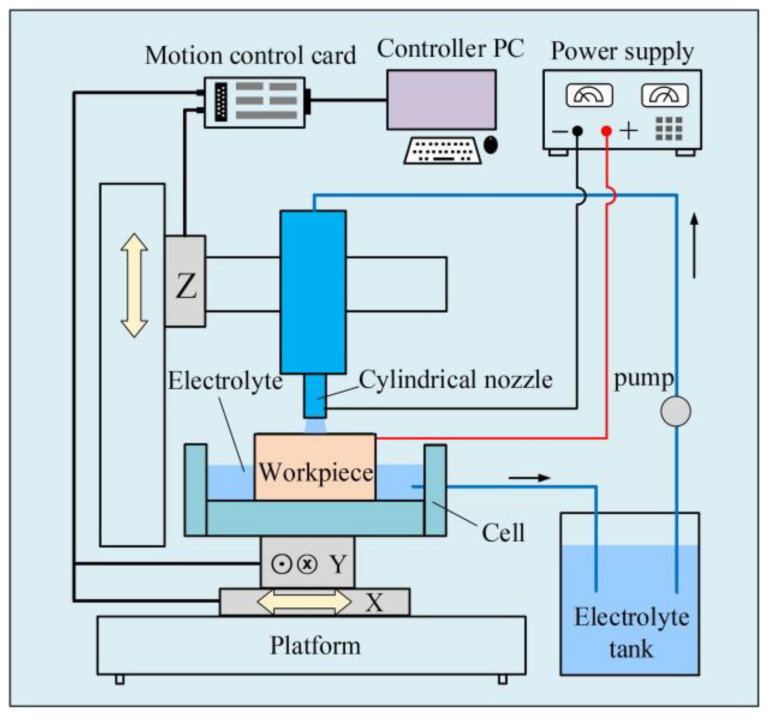
Schematic diagram of the experimental setup for processing amorphous alloy by Jet-ECM.

**Figure 2:**
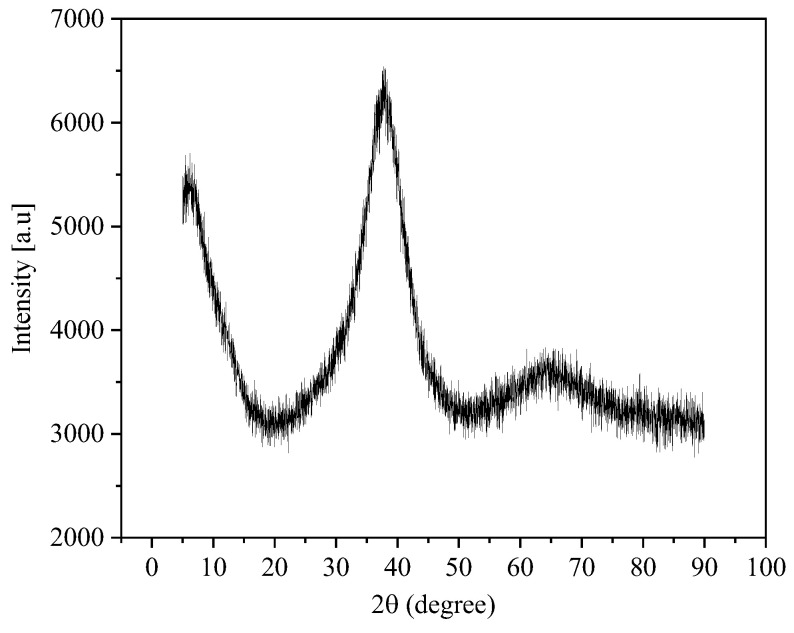
XRD patterns of Zr-based amorphous alloy (Zr_62.6_Ti_11_Cu_13.2_Ni_9.8_Be_3.4_).

The experiment setup for Jet-ECM of amorphous alloys is schematically illustrated in Figure 1. The micro-pit experiment was performed on the machine depicted in this schematic diagram. The electrolyte jet nozzle (SUS304, ID 220 ± 2 μm, OD 450 ± 3 μm) was installed on the *z*-axis of an X-Y-Z motion platform with a displacement accuracy of ±0.1 μm. The Zr-based amorphous alloys used in this paper were manufactured by PESHING NEW METAL, and the characteristics and elemental content of the material are presented in Table 1 and Table 2, respectively. The Zr-based AA (Zr_62.6_Ti_11_Cu_13.2_Ni_9.8_Be_3.4_) workpiece (20 mm (W) × 20 mm (L) × 2 mm (T)) was installed in an electrolytic cell, which was placed on the XY horizontal table. The XRD spectrum of the selected Zr-based amorphous alloy is shown in Figure 2. The positioning and moving of the nozzle and workpiece and the adjustment of the anode–cathode spacing were realized by moving the three axes of the platform. To reduce the oscillation of the electrolyte jet, the pressure applied to the electrolyte jet was produced by a constant high-pressure nitrogen gas tank, and the flow rate of the electrolyte jet was controlled at approximately 10 m/s. A DC power supply (IT-N6952, ITECH, Nanjing, China) was used to provide electric current for Jet-ECM.

An X-ray diffractometer (SmartLab SE, Rigaku, Japan) was used to characterize the degree of crystallization before and after the experimentation with a scanning range of 5–90°, a scanning rate of 5 °/min, and a scanning step size of 0.02°. Prior to the experimentation, the amorphous alloy sample was sequentially processed by polishing and ultrasonic degreasing cleaning and then dried in the air.

**Figure 3:**
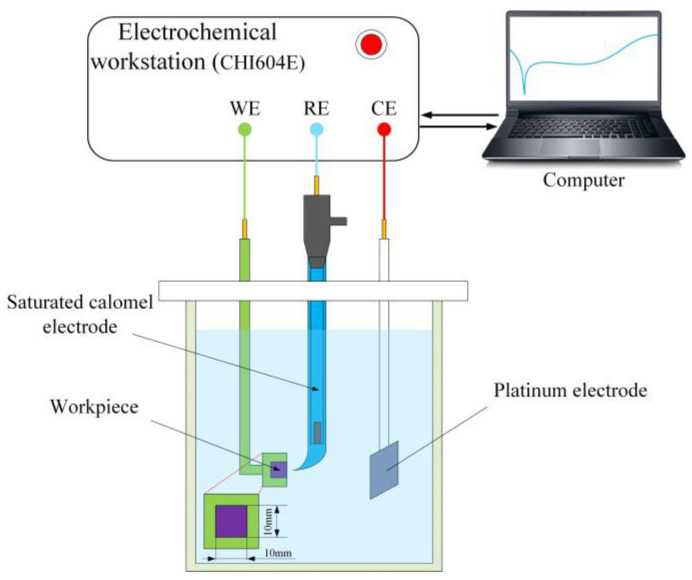
Schematic of the device used to measure polarization curves and EIS.

Electrochemical dissolution characteristics of amorphous alloys were analyzed using an electrochemical workstation (ChI604E, CH Instruments, Shanghai, China). As shown in Figure 3, the electrochemical tests were carried out in a standard three-electrode system in which platinum was used as the counter electrode (CE), calomel electrode as the reference electrode (RE), and the Zr-based amorphous alloy specimen (10 mm × 10 mm × 10 mm) as the working electrode (WE) with only 1 cm^2^ of its surface exposed to the electrolyte. The reference electrode (RE) and saturated calomel electrode (SEC) were connected to the electrolyte via an L-shape salt bridge filled with potassium chloride saturated solution and agar to reduce the liquid junction potential. Four typical electrolytes (1 M NaCl, 1 M HCl, 0.5 M H_2_SO_4_, and 1 M NaNO_3_) were intentionally selected to systematically assess the electrochemical dissolution characteristics of the Zr-based amorphous alloys. Before the electrochemical measurement, the samples to be examined were immersed in the electrolyte for 30 min. The open circuit potential (OCP) values were monitored to wait for a stable surface state. Polarization curve tests of the samples were performed with a potential range of −1–3 V and a sweep speed of 1 mV/s. Each selected electrolyte solution was tested at least three times, and the tested samples were immediately post-treated via ultrasonic cleaning.

Surface morphology and microstructural features of the samples before and after electrochemical testing and processing were analyzed using an ultra-deep field optical microscope (VHX2000, Keyence, Osaka, Japan) and a field emission scanning electron microscope (Merlin Compact, Zeiss, Jena, Germany). Electrochemical impedance spectroscopy (EIS) measurements were conducted in the frequency range from 10^−2^ to 10^5^ Hz. EDS analysis was performed before and after processing using a field emission scanning electron microscope (Merlin Compact, Zeiss, Germany). A laser confocal microscope (OLS5100, Olympus, Tokyo, Japan) was used to observe the morphological characteristics and to measure the surface roughness of the machined samples.

## 3. Results and Discussion

### 3.1. Electrochemical Characteristic Analysis of Zr-Based Amorphous Alloys

#### 3.1.1. Electrochemical Impedance Spectroscopy (EIS) Analysis

Previous studies [30] have shown that, compared to the acidic and neutral solutions frequently used for electrochemically machining AA, the alkaline solutions such as NaOH electrolytic will generate more insoluble electrolytic products, which seriously deteriorates the processing stability. Therefore, in this study, the acidic and neutral electrolyte was selected to evaluate. The selected electrolytes are 1 M NaCl, HCl, NaNO_3_, and 0.5 M H_2_SO_4_ solutions.

**Figure 4:**
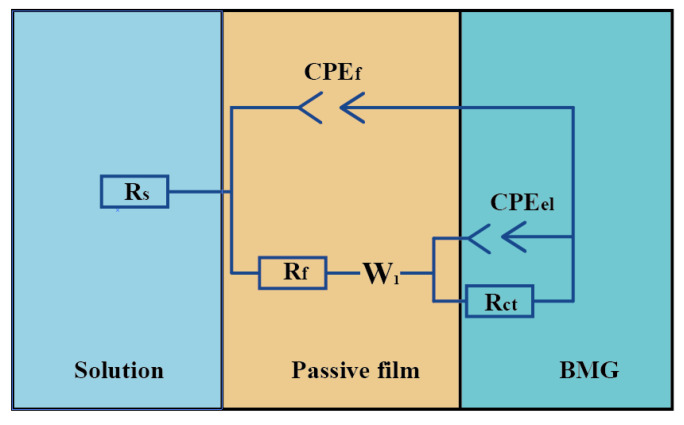
Equivalent electric circuit for electrochemically dissolving the amorphous alloys.

**Figure 5:**
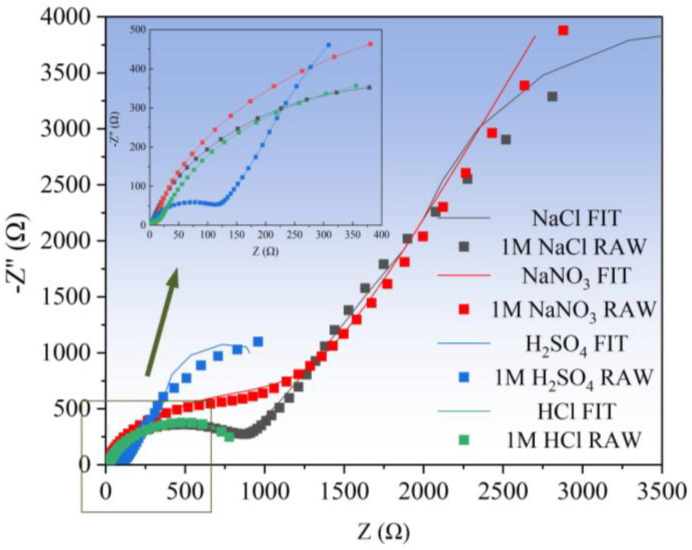
Nyquist plots obtained by using different electrolytes to process the Zr-based AA.

The electrochemical dissolution characteristics of Zr-based amorphous alloys were first analyzed by measuring their AC impedance spectra in the above-mentioned four solutions, and then their corrosion resistance was examined. The equivalent circuit shown in Figure 4 was adopted for EIS simulation. In this figure, R_s_ represents the solution resistance, R_f_ and CPE_f_ are the resistance and capacitance of the passive film, respectively, R_ct_ and CPE_el_ are the charge transfer resistance together with a double layer capacitance, and W_1_ denotes the Warburg impedance, which was introduced to improve the numerical fitting of the EIS results. Figure 5 presents the Nyquist diagram and its simulation curve of Zr-based amorphous alloy. The line and scatter plots represent the simulated data and measured data, respectively. It can be seen from Figure 5 that the radius of the circle of the Nyquist curve is the largest in NaNO_3_, the smallest in H_2_SO_4_, and is almost overlapped in HCl and NaCl, which are in between the above two extremes. This indicates that the corrosion resistance of the Zr-based amorphous alloys in the acidic solutions is much weaker. Therefore, the acidic solutions or the neutral solutions containing Cl^−^ ions are more suitable for electrochemical machining of the Zr-based AA.

#### 3.1.2. Electrochemical Polarization Characterization

**Figure 6:**
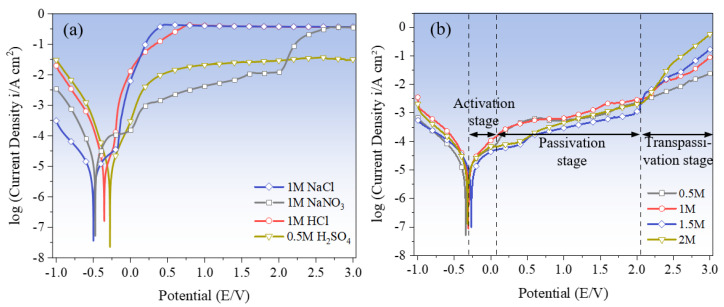
Polarization curves of the Zr-based AA (**a**) in 1 mol NaCl, HCl, H_2_SO_4_, and NaNO_3_ and (**b**) in different concentrations of NaNO_3_.

EIS impedance spectroscopy can reflect the impedance of Zr-based AA in the solution. To further analyze the specific corrosion resistance tendency and corrosion rate of Zr-based AA, polarization characteristics in the different electrolyte solutions were evaluated. Figure 6a shows the polarization curves of Zr_62.6_Ti_11_Cu_13.2_Ni_9.8_Be_3.4_ AA in 1 M NaCl, NaNO_3_, HCl, and 0.5 M H_2_SO_4_. Although the polarization processes of the AA in the different solutions all experience three stages, activation reaction stage, passivation stage, and transpassivation stage, the change trend of the respective polarization curve obtained is not the same. In NaNO_3_ solution, the initial corrosion potential is −0.47 V, and the corresponding current density is 8.01 A/cm^2^, then it enters the passivation state at 0.02 V and then enters the transpassivation state at approximately 2.02 V. In H_2_SO_4_ solution, the initial corrosion potential of Zr-based AA is −0.28 V, and the corresponding current density is 7.64 A/cm^2^, then it enters the passivation state at 0.03 V and then enters the transpassivation state at approximately 0.39 V. These changes are very similar to the results reported in references [2,3,4,5,6,7]. However, in NaCl and HCl solutions, the current density quickly rises to its highest value during a very short anodic passivation stage and then maintains a plateaus state, which indicates that the corrosion of the AA occurs. This finding is consistent with that reported by Green et al. [46]. It was also found that the Tafel curves measured at different NaNO_3_ concentrations show a significantly similar change trend, as shown in Figure 6b. This means that the concentration of the used electrolyte does not have a large impact on the polarization behaviors of the AA.

The specific parameters of the polarization curves in each solution are detailed in Table 3, where E_corr_, E_passi_, and E_trans_ are the initial corrosion potential, passivation potential, and transpassivation potential, respectively. I_corr_ and I_trans_, respectively, represent the initial corrosion current density and transpassivation current density. The magnitudes of the E_corr_ and I_corr_ reflect the corrosion tendency and the dissolution rate. Generally, higher E_corr_ and lower I_corr_ indicate that the amorphous alloy has higher stability and lower corrosion rate. As shown in Table 3, according to the order of E_corr_: NaCl < NaNO_3_ < HCl < H_2_SO_4_, which indicates that Zr-based AA dissolves more easily in NaCl solution first at the same voltage (−1–3 V) scan. On the other hand, the I_corr_ ordering is as follows: NaNO_3_ < H_2_SO_4_ < NaCl < HCl. This indicates that the Zr-based AA has the largest impedance value in NaNO_3_ solution, which corresponds to the smallest I_corr_ and the slowest corrosion rate, while the fastest corrosion rate is in HCl. Comprehensive analysis of E_corr_ and I_corr_ values shows that before the passivation of Zr-based AA starts, its corrosion tendency in acidic solution is smaller than that in neutral solution, but after the rupture of passivation film, Zr-based AA has the highest corrosion rate in HCl solution.

E_trans_ and I_trans_ indicate the voltage of corrosion required to fully enter the dissolved state and the transpassivation current density of complete dissolution. It was shown from Table 3 that the arrangement of E_trans_ and I_trans_ from small to large is as follows: NaCl < HCl < H_2_SO_4_ < NaNO_3_, H_2_SO_4_ < NaNO_3_ < NaCl < HCl, respectively. This shows that at the same voltage (−1–3 V) scan, the Zr-based AA is more likely to enter the overpassivation state in NaCl and HCl than in H_2_SO_4_ and NaNO_3_. In the fully dissolved state, the Zr-based AA has the fastest corrosion speed in the HCl solution and the slowest corrosion speed in the H_2_SO_4_ solution.

#### 3.1.3. Electrochemical Polarization Characterization

**Figure 7:**
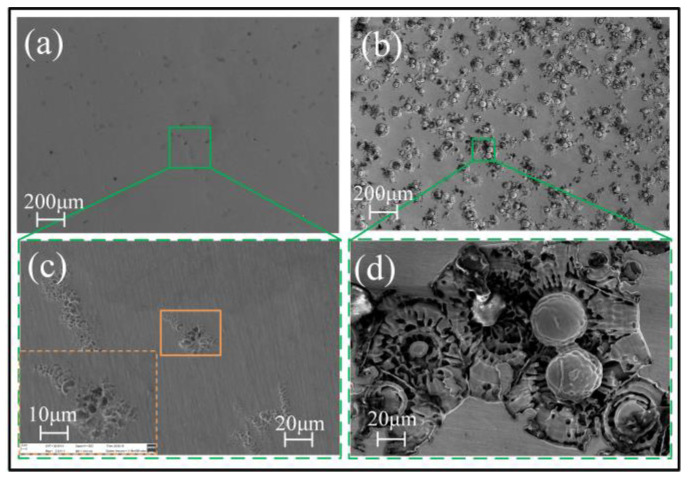
SEM images of corroded surface of the Zr-based AA after being tested in an electrode polarization state. (**a**,**c**) in 0.5 M H_2_SO_4_ solution (The orange dashed box is a 2.33KX magnification of the orange solid box); (**b**,**d**) in 1 M NaNO_3_ solution.

**Figure 8:**
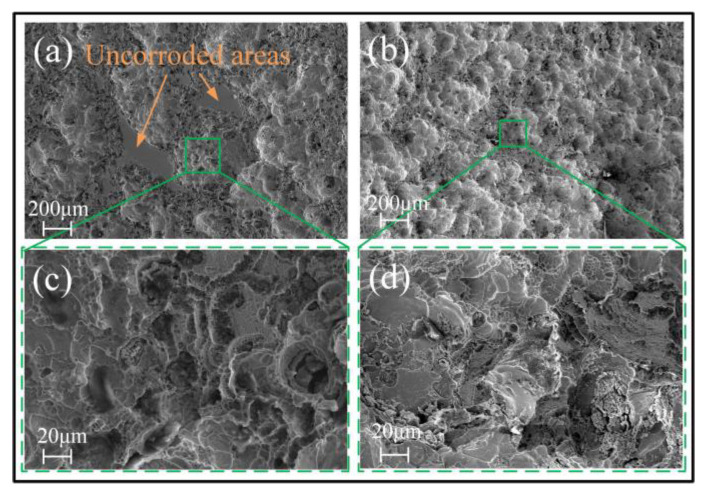
SEM images of the Zr-based AA corroded surface after polarization in 1 M HCl solution (**a**,**c**); 1 M NaCl solution (**b**,**d**).

Figure 7 and Figure 8 show the surface morphology of the samples after being tested in an electrode polarization state. It was found that the electrochemical dissolution characteristics of the Zr-based AA in the four solutions differ greatly. In the solution having oxidability, such as H_2_SO_4_ and NaNO_3_, the corroded surface has some sparsely scattered micro-pits, but they are shallow. However, in the two solutions containing Cl^−^, the corroded surface is very uneven, with a great number of mesoscale deep pits, and the surface material is significantly removed, as shown in Figure 8. Comparatively, the surface is corroded more greatly in the NaCl solution, showing a more fluctuant topography (shown in Figure 8b,d). The reasons for their great differences are as follows.

In the NaCl and HCl solutions, the newly formed passive film on the surface of Zr-based amorphous alloy is corroded and removed at an extremely fast rate due to the destructive effect of Cl^−^ in the electrolyte on the passive film. Previous studies [6,7,47] showed that the BeO in the natural passive film of the Zr_62.6_Ti_11_Cu_13.2_Ni_9.8_Be_3.4_ AA is preferentially hydrolyzed when it immerses in the Cl^−^-rich electrolytes without applying the polarization voltage. It was also found [8,9] that, in addition to the Be-rich locations on the surface of the amorphous alloy being the first to dissolve, Cu also easily reacts with Cl^−^ in the following way:

Cu + Cl^−^ = CuCl + e^−^ (1)

**Figure 9:**
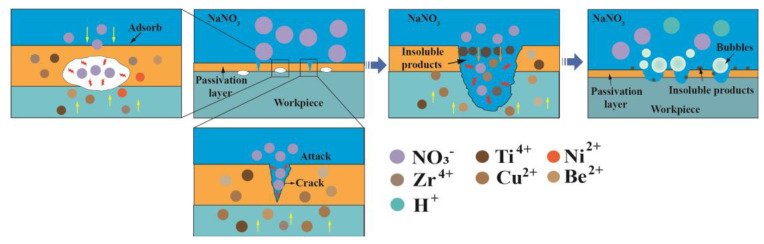
Pitting mechanism of Zr-based AA in the NaNO_3_ solution.

CuCl will be hydrolyzed to CuO or CuO_2_ in the subsequent reaction, which accelerates the dissolution of the amorphous alloy. This is also the reason why large-area dissolution of Zr-based AA occurs in the Cl^−^-containing electrolytes. Accordingly, a serious stray dissolution phenomenon can be observed on the nonmachined areas when using Jet-ECM to fabricate the Zr-based AA with a Cl^−^-containing electrolyte. On the other hand, the Zr-based AA is susceptible to be oxidized in an oxygen-containing solution such as H_2_SO_4_ and NaNO_3_ electrolytes, and thus oxidation film and passivation film will rapidly form once the anodic current is applied. This non-conductive oxidation film and passivation film prevent material from corroding [48]. However, there are some differences in dissolution mechanisms of Zr-based AA between the oxygen-containing acidic solution (for example, H_2_SO_4_) and the natural oxygen-containing solution (for example, NaNO_3_). In the H_2_SO_4_ solution, there are very few areas electrochemically dissolved due to a very low conductivity of the solution and a strong oxidability, as shown in Figure 7a,c. Different from H_2_SO_4_, NaNO_3_ is a neutral salt, and thus there may be two possible dissolution mechanisms. One is the defect-inducing mechanism. It is considered that electrochemical dissolution will preferentially occur within the microcrack defects where the electrolyte can permeate into, as shown in Figure 9. The other is the ion difference-inducing mechanism. Higher-concentration ions can penetrate into the interior of the passivation film and then react with the elements within the film [49]. With the further expansion of the internal corrosion area, a number of corrosion pits form on the surface of the AA, accompanied by the generation of a lot of oxides surrounding the corroded areas, such as TiO_2_, ZrO_2_, and other oxides [50]. These oxidation products to some extent inhibit the material from further corroding. This shows that NaNO_3_ can bring double effective effects for Jet-ECM of AA: enhance machining accuracy and prevent over-corroding of the nonmachined areas.

Through the above comprehensive analysis, it can be concluded that the more appropriate electrolyte for Jet-ECM of Zr-based AA is NaNO_3_ solution.

### 3.2. Fabrication of Micro-Dimples on the AA by Jet-ECM

#### 3.2.1. Effect of Electrolyte Compositions

**Figure 10:**
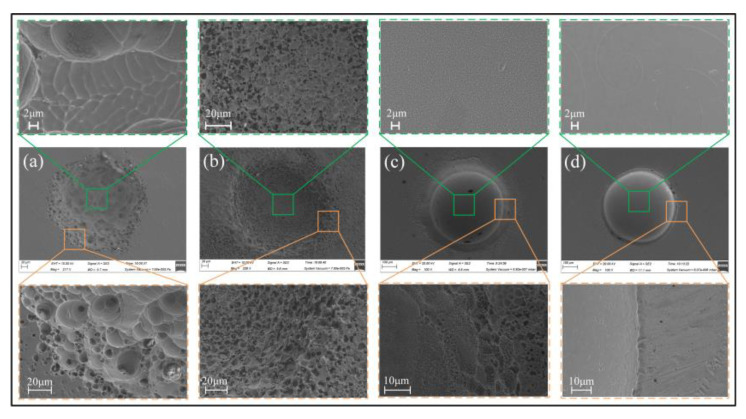
Micro-dimples processed by Jet-ECM with different electrolyte solutions. (**a**) 1 M HCl (Mag 217×); (**b**) 0.5 M H_2_SO_4_ (Mag 226×); (**c**) 1 M NaCl (Mag 100×); (**d**) 1 M NaNO_3_ (Mag 100×).

**Figure 11:**
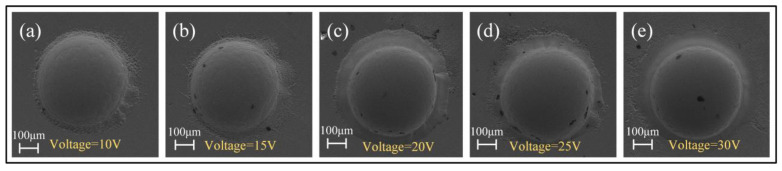
(**a**–**e**) Micro-dimples obtained at the applied voltages of 10–30 V in 1 M NaCl solution.

Figure 10 shows the morphology of the micro-dimples fabricated by Jet-ECM with 1 M HCl, 1 M NaCl, 1 M NaNO_3_, and 0.5 M H_2_SO_4_ solutions. The process parameters and conditions used are shown in Table 4. It can be seen from Figure 10 that it is difficult to form regular micro-dimple structures using both H_2_SO_4_ and HCl electrolytes (Figure 10a,b). The boundary areas of the machined micro-dimples are blurred and rough, and the inner area is also very rough, displaying densely distributed and dispersed pits of different sizes. In contrast, the micro-dimples processed based on NaCl and NaNO_3_ solution show a clearer and more regular boundary, the interior of the micro-dimples is relatively smooth, and the stray corrosion in the area outside the pit boundary is relatively light, as shown in Figure 10c,d. Further studies showed that compared with NaCl solution (as shown in Figure 11), the fabricated micro-dimples obtained with NaNO_3_ solution (as shown in Figure 10d) have a more regular profile, higher geometric dimensional accuracy, and smoother surface. These differences in morphological characteristics and dimensional accuracy of the machined micro-dimples are significantly due to the differences in the electrochemical characteristics of the Zr-based AA in the selected electrolyte. These findings indicate that the NaNO_3_ electrolyte is more suitable for the Jet-ECM of AA than other types of electrolytes.

**Figure 12:**
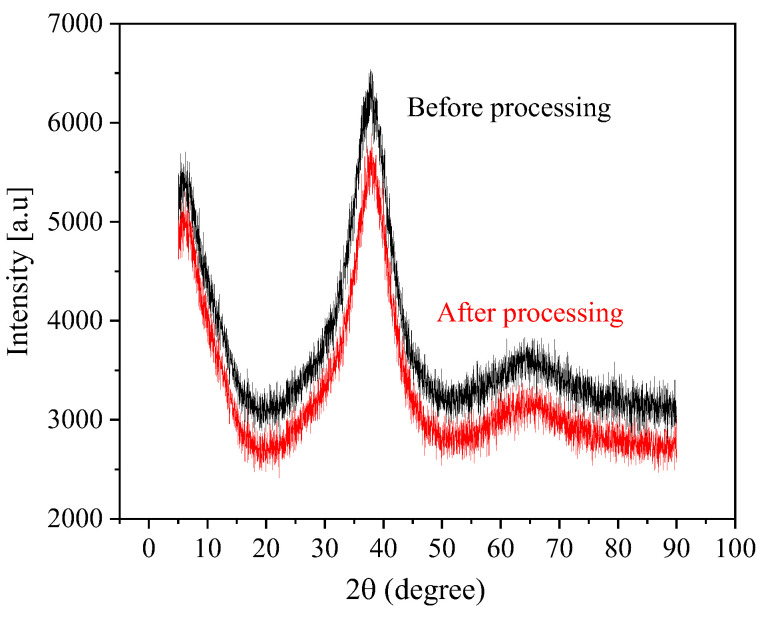
XRD patterns of the Zr-based AA before and after processing in the NaNO_3_ solution.

The machined AA samples were analyzed by X-ray diffraction, and the obtained XRD comparison charts of the Zr-based AA before and after electrochemical processing in the NaNO_3_ were achieved. It can be seen from Figure 12 that there are two dispersion peaks at 5° and 40°. It shows that the Zr-based AA is not crystallized before and after processing, which indicates that the Jet-ECM does not change the crystal structure of the AA.

#### 3.2.2. Effect of Electrolyte Concentration

**Figure 13:**
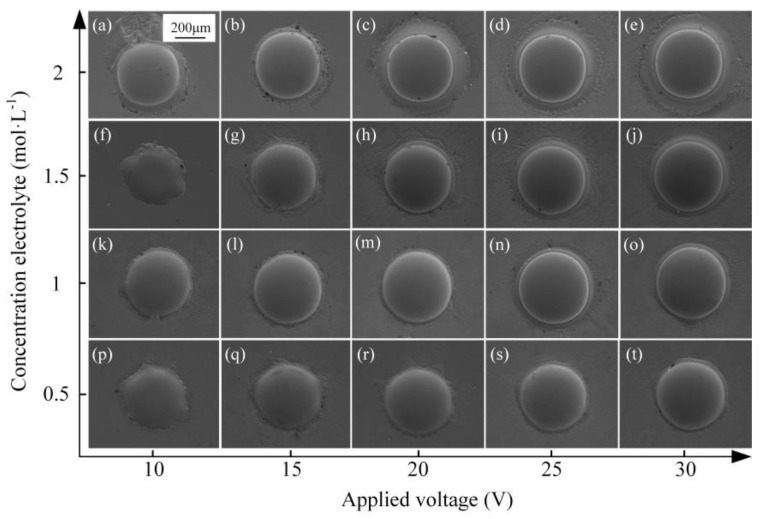
(**a**–**t**) Micro-dimples obtained using 0.5–2 M NaNO_3_ solutions and the applied voltage of 10–30 V.

**Figure 14:**
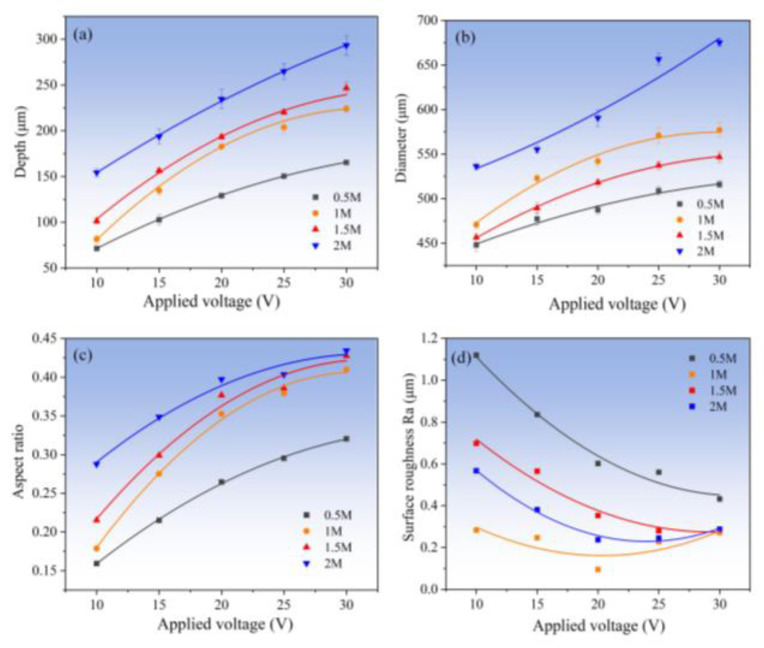
Change in geometric dimensional features (diameter, depth, and aspect ratio) of the machined micro-dimples with the different applied voltage and at different concentrations. (**a**) depth; (**b**) diameter; (**c**) aspect ratio; (**d**) surface roughness.

In order to determine the appropriate concentration of the used NaNO_3_ solution for the Jet-ECM of AA, the fabrication experimentation using varied concentrations of NaNO_3_ solution was carried out. The machined dimples are shown in Figure 13. In general, when the electrolyte concentration is relatively high, such as 1 M and 1.5 M, the micro-dimples’ profiles are more regular and their surfaces are smoother, but when the concentration is too high (such as 2 M) or too low (such as 0.5 M), the processed micro-pits are all not ideal. For example, when the concentration is 0.5 M, the acceptable micro-dimple structure can be obtained only at very high voltages of 15–20 V (Figure 13s,t), and when the concentration is 2 M, seriously stray corrosion at the boundaries can be observed in all the machined micro-dimples regardless of the applied inter-electrode voltages (Figure 13a–e). Figure 14 shows the changes of the geometric dimensions of the machined Zr-based AA micro-dimples fabricated at different concentrations of NaNO_3_ solution. It is shown that, the higher the concentration of NaNO_3_, the larger the diameter of the micro-dimples and the deeper the micro-dimples. This is mainly because a higher concentration means a higher conductivity of the electrolyte, and correspondingly, the higher concentration electrolyte will have a smaller interelectrode resistance, giving rise to a greater actual current density under a given interelectrode voltage. Therefore, more materials are dissolved and removed within a given processing time, thus generating deeper dimples. However, in effect, too high of a concentration cannot achieve overall desirable machining effects including surface quality, geometrical profile, aspect ratio, dimensional accuracy, etc., as shown in Figure 14. Comparatively, a moderately high concentration, i.e., 1 M, is more suitable. Therefore, 1 M NaNO_3_ electrolyte was selected in the following studies.

**Figure 15:**
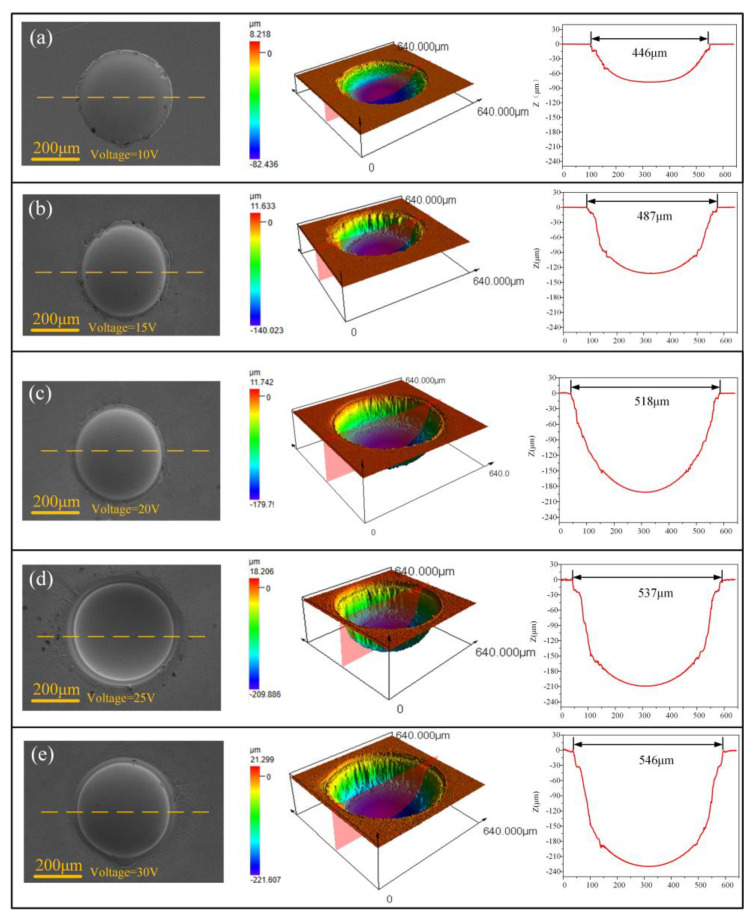
(**a**–**e**) The geometrical profile and surface morphologies of the micro-dimples fabricated with the parameters of 1 M NaNO_3_, 15 s processing time, and 10–30 V applied voltage.

Further studies showed that the applied voltage also has some effects on the surface morphologies and geometrical profile of the fabricated micro-dimples, although the same concentration of electrolyte was used, as shown in Figure 15. Generally, higher voltage produces deeper and larger dimples. However, the over-corrosion phenomenon at the boundary of the machined micro-dimples undergoes a vigorous fluctuation rather than a unidirectional change with increasing the applied voltage. That is, it transforms from heavy to slight and then to heavy. It was found that the use of moderately high voltage of 20 V can give rise to a considerably satisfactory machining effect. In such a case, the fabricated micro-dimples are very round and regular in shape and are very smooth in surface (surface roughness is down to Ra95 nm), showing little over-corrosion at their boundary. Therefore, 20 V is considered the most appropriate voltage value for Jet-ECM of the Zr-based AA.

### 3.3. Fabrication of Micro-Dimples on the AA by Jet-ECM

**Figure 16:**
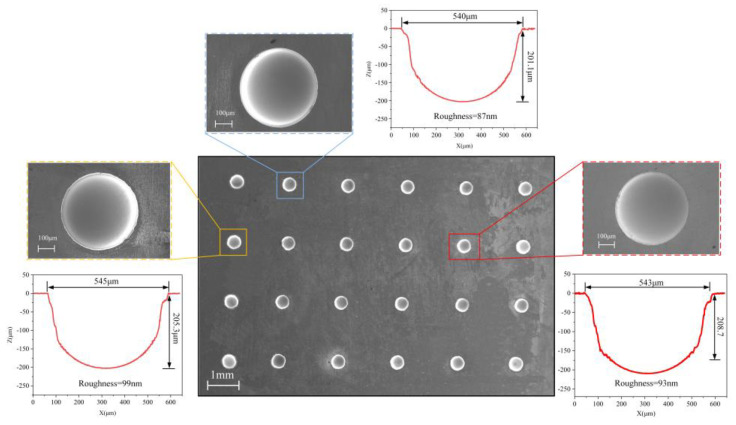
The arrayed micro-dimples fabricated by Jet-ECM using optimized processing parameters.

**Figure 17:**
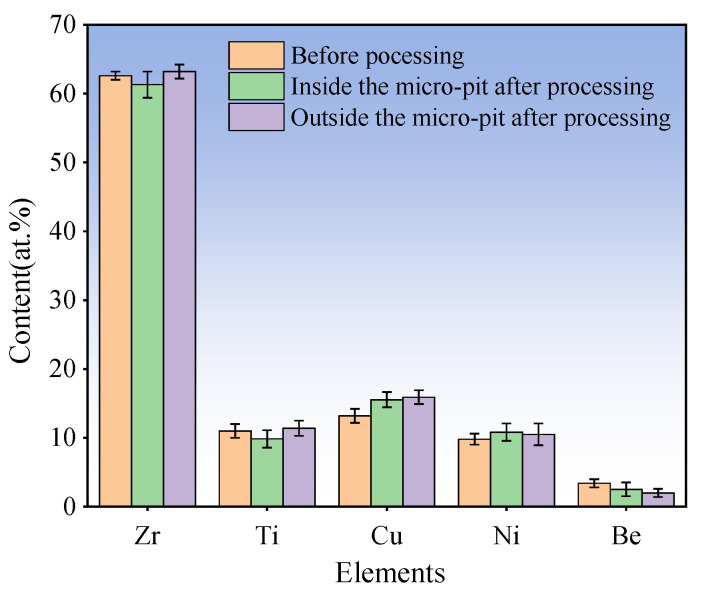
EDS results of Zr_62.6_Ti_11_Cu_13.2_Ni_9.8_Be_3.4_ AA before and after Jet-ECM.

As shown in Figure 16, the arrayed micro-dimples on the Zr_62.6_Ti_11_Cu_13.2_Ni_9.8_Be_3.4_ Zr-based AA surface machined by Jet-ECM are obtained with the following processing parameters optimized by the above-mentioned experimentation: 1 M NaNO_3_, 20 V voltage, 200 μm IEG, 15 s processing time, and 10 m/s impinging electrolyte flow rate. Three micro-dimples were randomly selected for the evaluation of the machining effect. It was found that the machined arrayed micro-dimples have a significantly high geometrical dimension consistence in diameter and depth sizes. Their depths are 206 ± 0.93 μm, and their diameters are 542.6 ± 2.3 μm, both showing very small dimension difference. Furthermore, the surface roughness of the machined arrayed micro-dimples is also very small, showing a mirror-like surface feature, with Ra being less than 100 nm. These findings indicate that Jet-ECM can achieve mirror-like micro-dimple structures with a significantly high geometrical consistency. Further tests of the machined surface using EDS demonstrated that there was little difference in the compositions of the surface material between the machined areas and unmachined areas, as shown in Figure 17. This indicates that the Jet-ECM processing hardly changes the compositions and microstructures of the Zr-based AA, and Jet-ECM is a favorably desirable microfabrication process to process the hard-to-cut Zr-based AA.

## 4. Conclusions

In this study, Jet-ECM was proposed to microfabricate Zr-based AA due to its low-temperature working environment and desirable material-removal mechanism. Optimal process parameters and conditions for the Jet-ECM of Zr-based AA were selected by studying the electrochemical characteristics of the Zr_62.6_Ti_11_Cu_13.2_Ni_9.8_Be_3.4_ material and surface morphologies and geometrical dimensional changes of the machined micro-dimples. Then, the feasibility of microfabricating arrayed structures on the Zr-based AA was evaluated. Some conclusions are drawn as follows.

1. Oxygen-containing electrolytes such as NaNO_3_ and H_2_SO_4_ are more suitable for the microfabrication of the Zr-based AA by Jet-ECM than chloride-containing electrolytes such as HCl and NaCl, and the NaNO_3_ electrolyte is the most desirable due to its high oxidization and electrochemical neutrality.
2. The material removal of Zr-based AA during Jet-ECM probably follows two mechanisms (defects-inducing mechanism and ion difference-inducing mechanism). Compared with other electrochemical processes, Jet-ECM is more advantageous due to the use of significantly high-speed electrolyte supply.
3. The arrayed micro-dimples with mirror-like surface features were fabricated on the surface of Zr-based AA using Jet-ECM, employing optimized processing parameters: 1 M NaNO_3_, 20 V voltage, 200 μm IEG, 15 s processing time, and 10 m/s impinging electrolyte flow rate. Their depths and diameters measure 206 ± 0.93 μm and 542.6 ± 2.3 μm, respectively, with Ra values less than 100 nm. Jet-ECM is a potentially competitive microfabrication process for Zr-based AA and can achieve mirror-like surface precision microstructures without being crystallized.

## Figures and Tables

**Table 1:** Material characteristics of the Zr-based AA.

Parameter	Property or Value
Density (g·cm^−3^)	6.12
Hardness (HV)	568–619
Poisson ratio	0.30
Specific conductance (MS/m)	0.52–0.53
Thermal conductivity (W·m^−1^·K^−1^)	4.601

**Table 2:** Main components of Zr-based AA.

**Element**	Zr	Ti	Cu	Ni	Be
**Element content (at.%)**	62.6%	11%	13.2%	9.8%	3.4%

**Table 3:** Corrosion parameters of the Zr-based AA samples after polarization in the different solutions.

Parameters	NaNO_3_	HCl	H_2_SO_4_	NaCl
E_corr_ (V)	−0.47	−0.35	−0.28	−0.50
E_passi_ (V)	0.02	−0.18	−0.05–0.25	−0.19–0.10
E_trans_ (V)	2.02	0.21	0.39	0.14
I_corr_ (A·cm^2^)	−0.81	−6.79	−7.64	−7.44
I_trans_ (A·cm^2^)	−1.91	−0.57	−2.42	−1.05

**Table 4:** Machining parameters and conditions for fabricating micro-dimples using Jet-ECM.

Machining Parameter	Property or Value
Nozzle material	SUS 304
Nozzle diameter/μm	ID: 220 ± 2; OD: 450 ± 3
Electrolyte composition	NaNO_3_, H_2_SO_4_, HCl and NaCl
Concentration/(mol/L)	0.5 or 1
Machining gap/μm	200
Machining voltage/V	10, 15, 20, 25, 30
Electrolyte velocity (m/s)	10
Machining time/s	15
Temperature of electrolyte/°C	25 ± 2

## Data Availability

Data are contained within the article.
